# CWF19L1 promotes T-cell cytotoxicity through the regulation of alternative splicing

**DOI:** 10.1016/j.jbc.2024.107982

**Published:** 2024-11-13

**Authors:** Yuqi Zhang, Jingjing Yi, Gaigai Wei, Tingrong Ren, Haiping Zhao, Huiling Zhang, Hui Yang, Duanwu Zhang

**Affiliations:** 1 Children′s Hospital of Fudan University, National Children′s Medical Center, Shanghai Key Laboratory of Medical Epigenetics, International Co-laboratory of Medical Epigenetics and Metabolism, Ministry of Science and Technology, Institutes of Biomedical Sciences, Fudan University, Shanghai, China; 2 Department of Neurosurgery, Huashan Hospital, Institute for Translational Brain Research, Fudan University, Shanghai, China; 3 State Key Laboratory of Medical Neurobiology and MOE Frontiers Center for Brain Science, Shanghai Medical College, Fudan University, Shanghai, China

**Keywords:** CWF19L1, splicing, splicing factor, alternative splicing, spliceosome, snRNP, t cell, cytotoxicity, cytokine, antitumor immunity

## Abstract

Effective cancer immunotherapy relies on enhancing the host's immune response, particularly by boosting T cell-mediated cytotoxicity against tumor cells. In this study, we identify CWF19-like cell cycle control factor 1 (CWF19L1) as a novel splicing regulator that enhances T cell-mediated cytotoxicity. CWF19L1 interacts prominently with key splicing factors within the nucleus, including components of the U5 small nuclear ribonucleoprotein and the pre-mRNA processing factor 19 (PRPF19) complex. Deficiency of CWF19L1 disrupts alternative splicing of immune-related genes, resulting in diminished expression of cytotoxic molecules. Furthermore, CWF19L1 plays a critical role in promoting T cell-mediated antitumor responses by upregulating the expression of effector cytokines. Our findings unveil previously undocumented functions of CWF19L1 in alternative splicing and its involvement in the regulation of antitumor immunity, highlighting its potential as a therapeutic target for novel cancer immunotherapies.

Pre-mRNA splicing, catalyzed by the spliceosome, represents a pivotal process in gene expression regulation. Following transcription from DNA, pre-mRNA undergoes splicing to remove introns, generating mature mRNA that directs protein synthesis during translation (1, 2). Alternative splicing further diversifies mRNA transcripts, enabling the translation of a single gene into multiple protein isoforms with distinct functions (3). The spliceosome is a dynamic ribonucleoprotein complex composed of U1, U2, U5, and U4/U6 snRNPs, assembled through interactions with small nuclear RNAs (4). The spliceosome undergoes extensive conformational changes facilitated by *trans*-acting proteins and DEAx/H-box adenosine triphosphatases (5). Recent studies highlight the critical roles of spliceosome components such as polyglutamine binding protein 1 (PQBP1), CD2 cytoplasmic tail binding protein 2 (CD2BP2), and small nuclear ribonucleoprotein U5 subunit 200 (SNRNP200) in normal immune responses, underscoring the interplay between splicing and immunity (6, 7, 8, 9, 10, 11).

Cytotoxicity, or cell-mediated killing, encompasses mechanisms whereby immune cells eliminate target cells. It is categorized into basic (level I) cytotoxicity mediated by macrophages and inducible (level II) cytotoxicity requiring specific activation (12). Level I involves M1 macrophages activated by interferon-γ and microbial products, and neutrophils exerting cytotoxic effects *via* oxygen radicals. Level II includes cytotoxic lymphocytes (CTLs), natural killer (NK) cells, and antibody-dependent cell-mediated cytotoxicity (13). These cells use various mechanisms such as perforin-granzyme release and death receptor-mediated apoptosis to eliminate target cells, crucial for antitumor immunity in the tumor microenvironment (14, 15, 16, 17).

CWF19L1, initially identified as a hereditary ataxia-related molecule, has recently garnered attention for its effects in cancer (18, 19, 20, 21, 22, 23, 24). In kidney renal clear-cell carcinoma, CWF19L1 may enhance cancer progression by increasing gene expression through RNA editing sites in the 3′ UTR and promoting tumor cell proliferation (25). Conversely, in glioblastoma, CWF19L1 has been identified as a favorable prognostic factor due to its role in inhibiting the transition from the G1 phase to the S phase of the cell cycle (26). These findings highlight CWF19L1′s diverse roles in cancer biology and its potential as a therapeutic target in cancer treatment. In addition to its association with hereditary ataxia and tumor cell proliferation, CWF19L1 is also implicated in mRNA processing, although its precise role in splicing remains unclear (27).

In this study, we reveal CWF19L1 as a novel splicing factor that regulates global alternative splicing and enhances CD8+ T cell-mediated cytotoxicity. We demonstrate interactions between CWF19L1 and key spliceosomal proteins, including elements of U5 snRNP and the pre-mRNA processing factor 19 (PRPF19) complex. Furthermore, CWF19L1 influences immune-related gene splicing, impacting the expression of cytotoxic molecules and effector cytokines. Our findings suggest CWF19L1 as a potential therapeutic target in cancer immunotherapy.

## Results

### CWF19L1 interacts with the U5 snRNP and the PRPF19 complex

**Figure 1:**
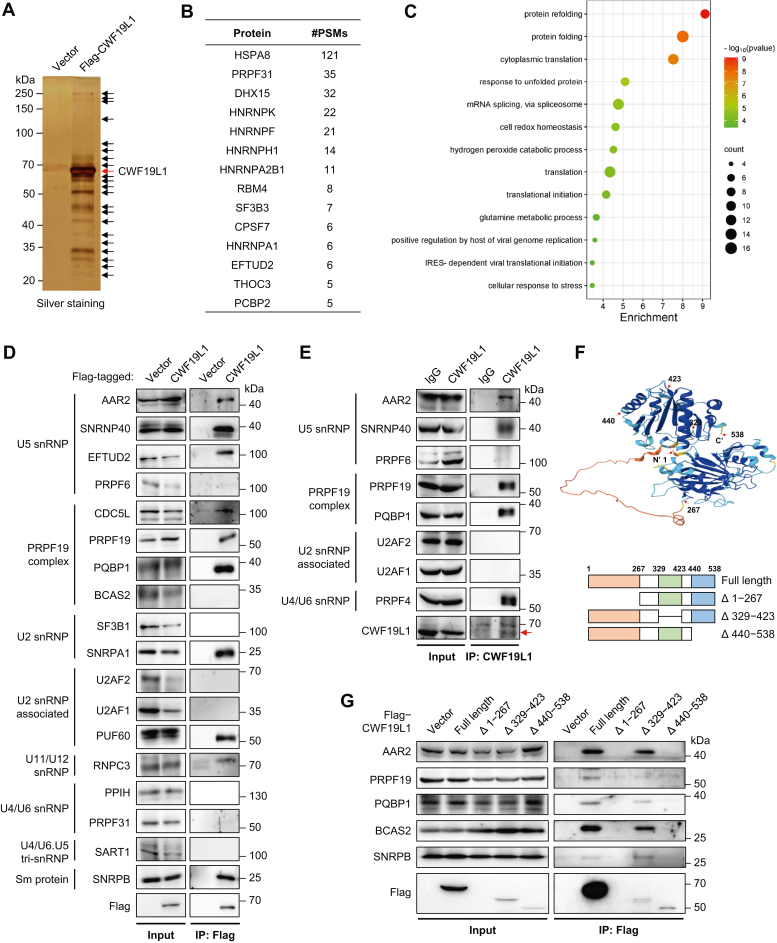
**CWF19L1 interacts with the components of spliceosomal complexes**. *A*, HEK293T cells were transfected with Flag vector or Flag-tagged CWF19L1. Cell lysates were immunoprecipitated using anti-Flag M2 beads and the bound proteins were analyzed by silver staining and mass spectrometry (LC-MS). *B*, the top splicing-related proteins interacting with CWF19L1 revealed by the Co-IP/MS. A comprehensive list of CWF19L1-interacting candidates is provided in Dataset S1. *C*, enrichment analysis of CWF19L1-associated proteins identified in the Co-IP/MS experiment. *D*, HEK293T cells were transfected with Flag vector or Flag-tagged CWF19L1. Cell lysates were immunoprecipitated using anti-Flag M2 beads and the bound endogenous proteins were analyzed by Western blot using the indicated antibodies. *E*, endogenous immunoprecipitation: A549 cell lysates were immunoprecipitated using IgG or anti-CWF19L1 antibody and analyzed by Western blot for the indicated proteins. The *red arrow* indicates the fraction of CWF19L1. *F*, CWF19L1 truncated mutants were designed and generated based on the AlphaFold prediction. Groups of α-helixes, β-sheets, and disordered regions were assigned into different mutants. *G*, HEK293T cells were transfected with Flag vector, Flag-tagged CWF19L1 or CWF19L1 truncated mutants. Cell lysates were immunoprecipitated using anti-Flag M2 beads and analyzed by Western blot with the indicated antibodies. IgG, immunoglobulin G.

According to the BioPlex interactome and STRING protein-protein interaction networks, CWF19L1 is predicted to be part of spliceosome complex (Fig. S1, *A*−*C*). Using coimmunoprecipitation combined with mass spectrometry, we identified dozens of splicing factors and regulators that interacted with CWF19L1 (Fig. 1, *A* and *B*). Consistently, Gene Ontology (GO) analysis of CWF19L1-interacting candidates showed that most of these proteins were involved in protein folding and mRNA splicing (Fig. 1*C* and Fig. S1*D*). The follow-up immunoblot analysis verified the interactions between CWF19L1 and spliceosome components, including U5 snRNP proteins (EFTUD2 and SNRNP40) and core subunits of the PRPF19 complex (PRPF19, CDC5L, and PQBP1). In contrast, interactions between CWF19L1 and U4/U6-specific proteins (PPIH and PRPF31) were hard to detect (Fig. 1*D*). Endogenous immunoprecipitation confirmed the interactions between CWF19L1 and the U5 snRNP and the PRPF19 complex (Fig. 1*E*). To define the structural domain in CWF19L1 responsible for its interaction with the spliceosome, we designed and used the truncated mutants of CWF19L1 based on the predictions from the AlphaFold (https://alphafold.ebi.ac.uk/) (Fig. 1*F*). Deletion of the middle domain (amino acids 329−423) did not affect the interaction of CWF19L1 with spliceosomal proteins. However, deletion of the amino terminus (amino acids 1−267) or the carboxy terminus (amino acids 440 − 538) destabilized CWF19L1 protein and dissipated the interactions (Fig. 1*G*). These results indicate that both the amino terminal and carboxy terminal regions of CWF19L1 are essential for its stability and its interaction with the spliceosome. Notably, CWF19L1 was mainly localized to the nucleoplasm, which harbors abundant spliceosomal proteins. However, colocalization of CWF19L1 and specific nuclear substructures such as Cajal body, nuclear speckle, nucleoli, promyelocytic leukemia-nuclear body, and paraspeckle were not detected (Fig. S2). Collectively, these findings suggest that CWF19L1 participates in mRNA splicing *via* interactions with spliceosomal complexes, in especial with U5 snRNP and the PRPF19 complex.

### CWF19L1 deficiency results in global splicing abnormalities

**Figure 2:**
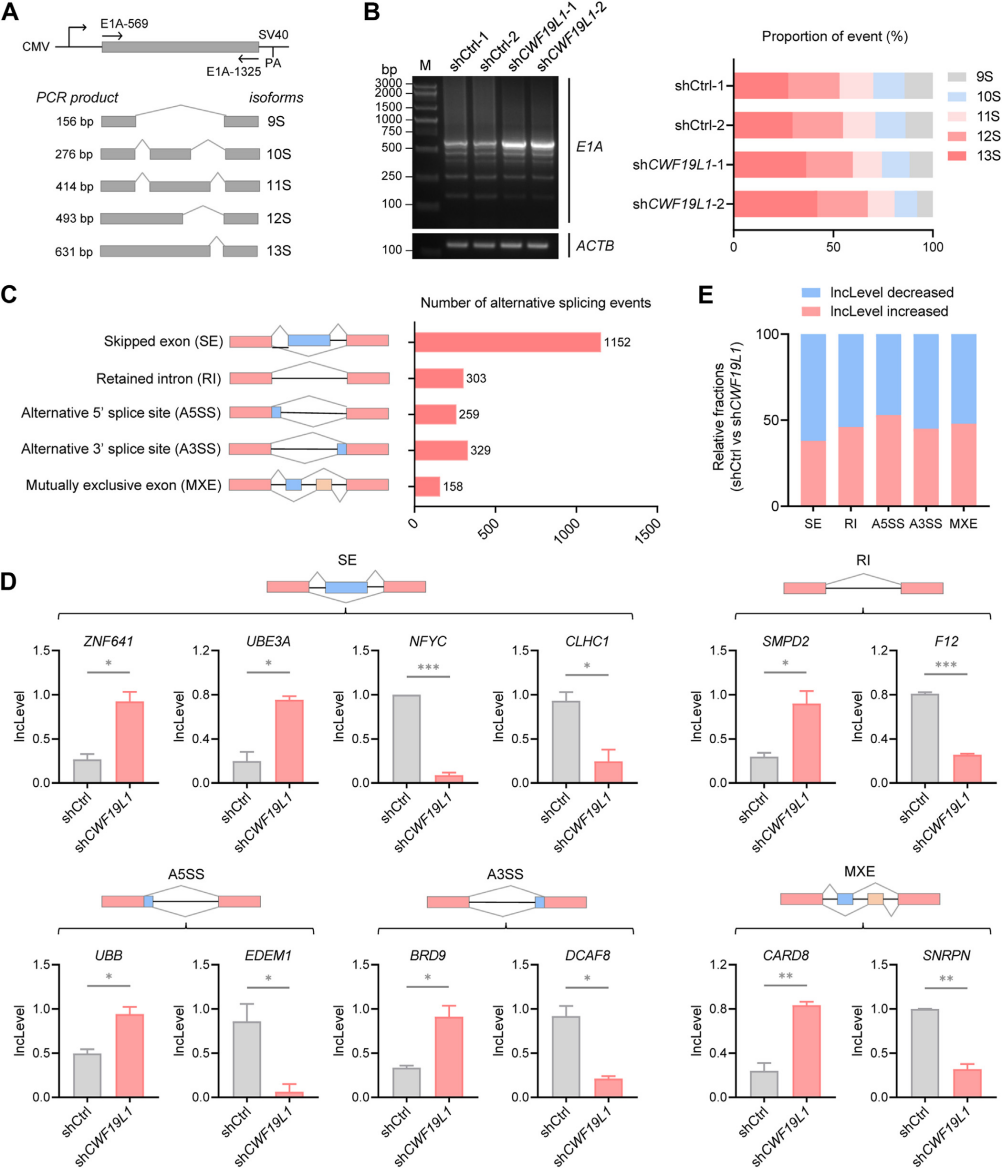
**CWF19L1 deficiency leads to alternative splicing defects**. *A*, diagram of the E1A reporter minigene (pCMV-E1A) illustrating the alternative splicing events that produce 13S, 12S, 11S, 10S, and 9S transcripts. The positions of primers used for RT−PCR analysis are indicated. *B*, RT−PCR analysis of E1A minigene. HEK293T cells infected with lentivirus encoding short-hairpin RNA (shRNA) against *CWF19L1* (sh*CWF19L1*) or nontargeting control shRNA (shCtrl). A representative gel analysis (*left*) and quantification of each splicing isoform by ImageJ from three independent biological replicates (*right*) are shown. *C*, number of alternative splicing events in CWF19L1-depleted Jurkat cells, revealed by RNA-seq. *D*, representative of various types of alternative splicing in CWF19L1-depleted Jurkat cells compared to control Jurkat cells. *p* values were calculated using an unpaired Student′s *t* test. Data are presented as mean ± SD (∗*p* < 0.05, ∗∗*p* < 0.01, ∗∗∗*p* < 0.001). *E*, relative proportions of increased and decreased inclusion levels induced by CWF19L1 deficiency in Jurkat cells.

To examine the potential role of CWF19L1 in mRNA splicing, we transiently transfected an E1A reporter plasmid as a minigene into control and *CWF19L1*-depleted HEK293T cell lines (Fig. 2*A* and Fig. S3*A*). As expected, CWF19L1 deficiency altered the splicing pattern of E1A minigene, in particular inhibiting selection of the most distal 5′ splice site, leading to a decline of 9S isoform and an increase of 13S isoform (Fig. 2*B*). To gain further insight into CWF19L1-regulated alternative splicing events, we performed RNA sequencing (RNA-seq) on *CWF19L1*-depleted and control Jurkat cells. Over 2000 alternative splicing abnormalities caused by CWF19L1 deficiency were identified, including skipped exons (SEs), retained introns (RIs), alternative 5′ splice sites (A5SSs), alternative 3′ splice sites (A3SSs), and mutually exclusive exons, among which the most frequently occurring were SEs (Fig. 2, *C* and *D*, and Dataset S2). Subsequent analysis revealed a predominance of negative regulation of alternative splicing events in response to CWF19L1 deficiency, as indicated by inclusion levels alterations (Fig. 2*E*). Collectively, these findings underscore that CWF19L1 deficiency induces extensive alterations in the global splicing landscape.

### CWF19L1 deficiency causes widespread alterations in gene expression

**Figure 3:**
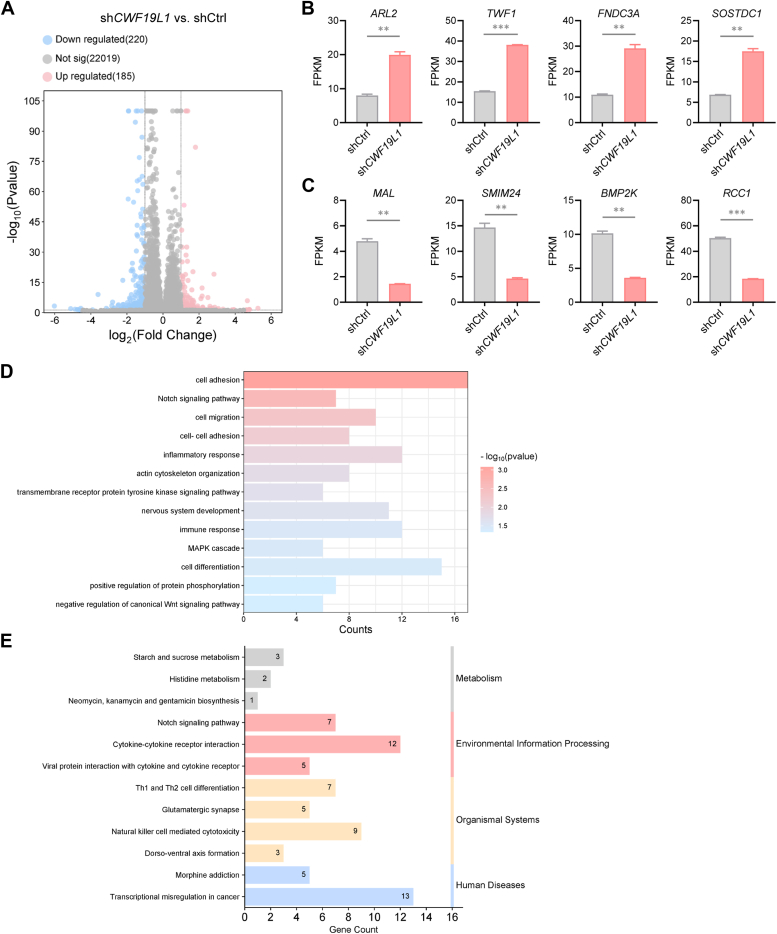
**CWF19L1 deficiency causes gene expression changes.***A*, volcano plot depicting the relative expression levels of genes upregulated and downregulated in CWF19L1-depleted *versus* control Jurkat cells. *Red* and *blue dots* indicate significantly upregulated and downregulated genes, respectively. Each dot represents an individual gene. *B* and *C*, representative genes that are upregulated (*B*) and downregulated (*C*) due to CWF19L1 deficiency. *p* values were calculated using an unpaired Student′s *t* test. Data are presented as mean ± SD (∗∗*p* < 0.01, ∗∗∗*p* < 0.001). *D* and *E*, GO biological process and KEGG pathway analyses of differentially expressed genes in CWF19L1-depleted *versus* control Jurkat cells. GO, Gene Ontology; KEGG, Kyoto Encyclopedia of Genes and Genomes.

We further investigated the impact of CWF19L1 deficiency on the transcriptome. The transcriptome profiling revealed misexpression of 405 genes among which 185 genes were upregulated while 220 genes were downregulated (Fig. 3, *A*−*C*, and Dataset S3). GO enrichment analysis highlighted that these genes were significantly associated with processes such as cell adhesion, cell differentiation, inflammatory response, and immune response (Fig. 3*D* and Fig. S3*B*). Moreover, these genes are involved in pathways including transcriptional misregulation in cancer, cytokine-cytokine receptor interaction, NK cell-mediated cytotoxicity and Th1 and Th2 cell differentiation (Fig. 3*E*). Intriguingly, analysis of gene expression patterns revealed that CWF19L1 was highly conserved across different species and prominently expressed in immune tissues including bone marrow, lymph node, thymus, and spleen (Figs. S4 and S5*A*). Additionally, CWF19L1 showed ubiquitous expression across most immune cells including T cells and NK cells (Fig. S5*B*). Thus, all these data indicate that CWF19L1 deficiency leads to transcriptome-wide changes in gene expression, particularly affecting immune-related genes.

### CWF19L1 deficiency induces splicing defects of immune-related genes

**Figure 4:**
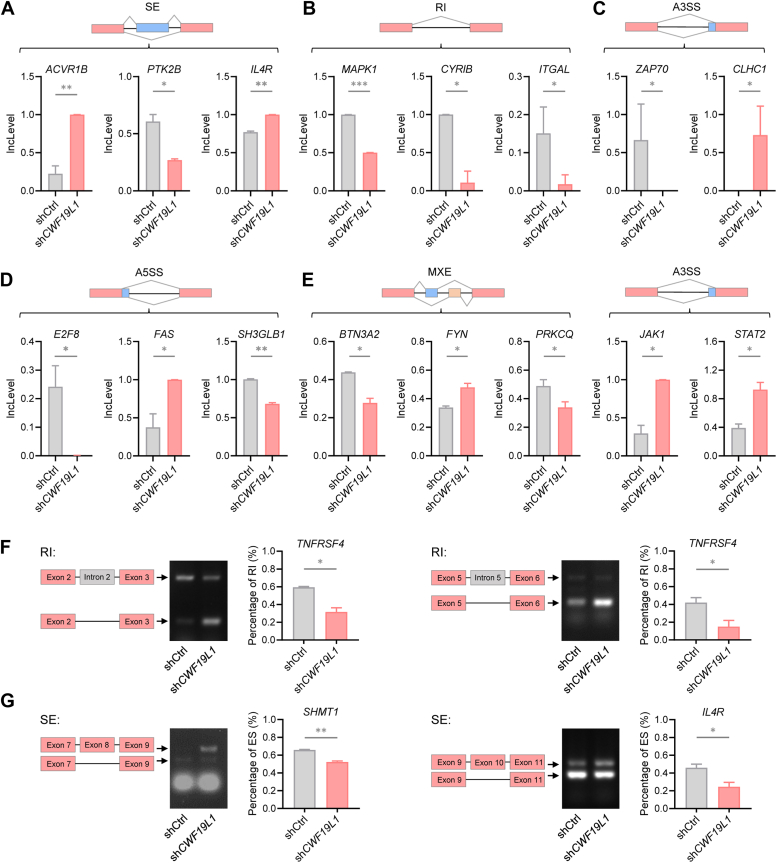
**CWF19L1 deficiency induces splicing abnormalities of immune-related genes**. *A*−*E*, representative of different types of alternative splicing events of immune-related genes in *CWF19L1* knockdown and control Jurkat cells. (*A*, skipped exons; *B*, retained introns; *C*, alternative 3′ splice sites; *D*, alternative 5′ splice sites; *E*, mutually exclusive exons). *p* values were calculated using an unpaired Student′s *t* test. Data are presented as mean ± SD (∗*p* < 0.05, ∗∗*p* < 0.01, ∗∗∗*p* < 0.001). *F* and *G*, RT-PCR and fragment analyses of alternative splicing in representative immune-related genes in CWF19L1-depleted and control Jurkat cells. The results for RI (*F*) and SE (*G*) are shown. A representative gel analysis (*left*) and quantification from three independent biological replicates (*right*) are shown. *p* values were calculated using an unpaired Student′s *t* test. Data are presented as mean ± SD (∗*p* < 0.05, ∗∗*p* < 0.01). RI, retained intron; SE, skipped exon.

To investigate the role of CWF19L1 in immune regulation, we assessed its impact on immune-related gene splicing. All types of alternative splicing abnormalities were observed in CWF19L1-deficiency cells (Fig. 4, *A*−*E*). RIs and SEs in representative immune-related genes in CWF19L1-deficient Jurkat cells were validated by RT-PCR (Fig. 4, *F* and *G*). Although SEs were predominant, Kyoto Encyclopedia of Genes and Genomes (KEGG) pathway analyses of different types of alternative splicing abnormalities showed that A3SSs and RIs were particularly associated with immune-related genes (Fig. 2*C* and Fig. S6, *A*−*F*). Collectively, these findings indicate that CWF19L1 deficiency disrupts immune gene splicing, highlighting its potential role in immune modulation.

### CWF19L1 deficiency affects immune-related gene expression

**Figure 5:**
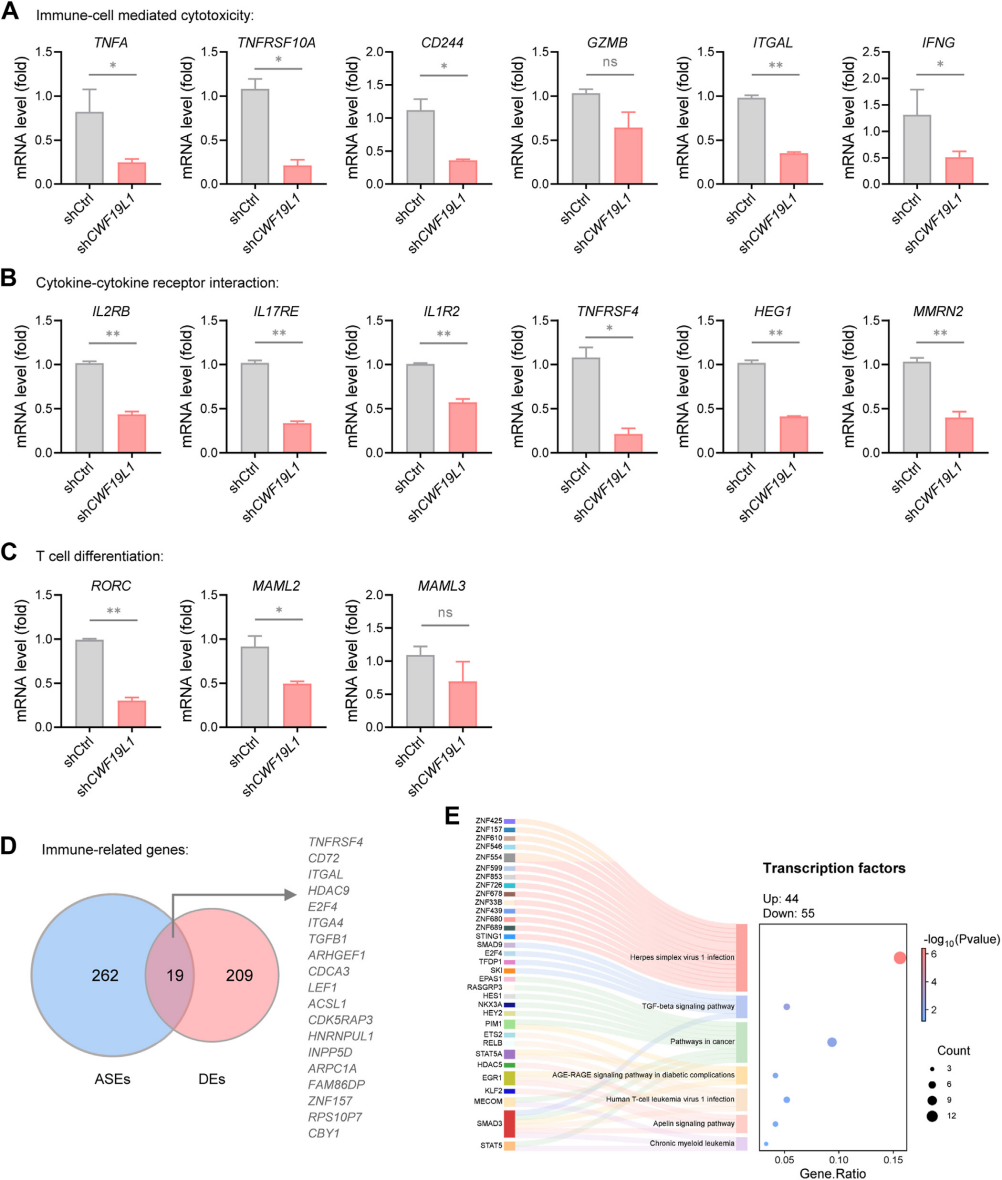
**CWF19L1 deficiency affects immune-related gene expression.***A*−*C*, reverse transcription quantitative polymerase chain reaction (RT−qPCR) analysis of genes associated with immune-cell-mediated cytotoxicity (*A*), cytokine-cytokine receptor interaction (*B*), and T cell differentiation (*C*) in CWF19L1-depleted and control Jurkat cells. *p* values were calculated using an unpaired Student′s *t* test. Data are presented as mean ± SD (∗*p* < 0.05, ∗∗*p* < 0.01). *D*, Venn diagram showing the overlap of genes between alternative splicing events (ASEs) and differentially expressed (DEs) immune-related genes in CWF19L1-deficient *versus* control Jurkat cells. *E*, KEGG enrichment analysis of differentially expressed transcription factors between CWF19L1-deficient and control Jurkat cells. KEGG, Kyoto Encyclopedia of Genes and Genomes.

Given the observed impact of CWF19L1 deficiency on immune-related gene splicing, coupled with GO enrichment and KEGG pathway analyses revealing its potential regulatory role in immune-related gene expression, we conducted quantitative real-time PCR to validate differential expression of immune-related candidates in Jurkat T cells (Fig. 5, *A*−*C*). Consistently, downregulation of genes involved in immune-cell mediated cytotoxicity, cytokine-cytokine receptor interaction, and T cell differentiation were verified in CWF19L1-deficient Jurkat cells. To investigate whether splicing defects contribute to altered immune gene expression, we conducted a comparative analysis of alternative splicing events and differential expressions in CWF19L1-deficient cells. We identified an overlap between aberrantly spliced immune-related genes and those with differential expression, suggesting that impaired splicing at least partially accounts for the decreased expression of immune-related genes (Fig. 5*D*). Additionally, we observed CWF19L1 deficiency was associated with altered expression of several transcription factors (Fig. 5*E*), which may also contribute to the altered expression of immune-related genes. Taken together, these findings suggest that CWF19L1 deficiency disrupts immune-related gene expression through both direct regulation of alternative splicing and modulation of upstream transcription factors.

### CWF19L1 enhances cytokine production in T cells upon stimulation

**Figure 6:**
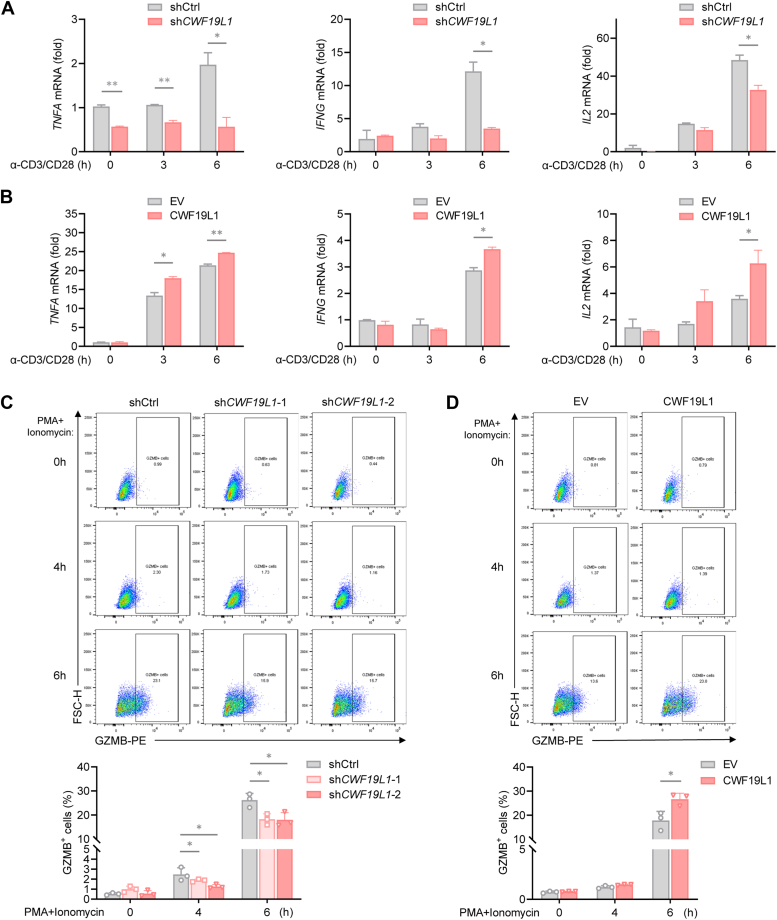
**CWF19L1 augments cytokine production in T cells**. *A*, reverse transcription quantitative polymerase chain reaction (RT−qPCR) analysis of lFN-γ, TNF-α, and GZMB expression in CWF19L1-depleted and control Jurkat cells stimulated with anti-CD3 and anti-CD28 antibodies for the indicated time periods. *p* values were calculated using an unpaired Student′s *t* test. Data are presented as mean ± SD (∗*p* < 0.05, ∗∗*p* < 0.01). *B*, RT−qPCR analysis of lFN-γ, TNF-α, and GZMB expression in CWF19L1-overexpressing and control Jurkat cells stimulated with anti-CD3 and anti-CD28 antibodies for the indicated time periods. *p* values were calculated using an unpaired Student′s *t* test. Data are presented as mean ± SD (∗*p* < 0.05, ∗∗*p* < 0.01). *C*, flow cytometric analysis of GZMB expression in CWF19L1-depleted and control Jurkat cells stimulated with 50 ng/ml PMA and 1 μg/ml ionomycin for the indicated time periods. *p* values were calculated using an unpaired Student′s *t* test. Data are presented as mean ± SD (∗*p* < 0.05). *D*, flow cytometric analysis of GZMB expression in CWF19L1-overexpressing and control Jurkat cells stimulated with 50 ng/ml PMA and 1 μg/ml ionomycin for the indicated time periods. *p* values were calculated using an unpaired Student′s *t* test. Data are presented as mean ± SD (∗*p* < 0.05). GZMB, granzyme B; IFN, interferon; PMA, phorbol 12-myristate 13-acetate; TNF, tumor necrosis factor.

Given that diminished expression of genes associated with immune functions was observed in CWF19L1-deficient cells, we sought to determine the effects of CWF19L1 on immune responses. Since differentially expressed genes induced by CWF19L1 deficiency were significantly associated with cytokine-cytokine receptor interaction and cytotoxicity, we assessed the role of CWF19L1 in T cell function. Upon activation by anti-CD3α/anti-CD28 antibodies, CWF19L1-deficient Jurkat cells exhibited impaired effector function manifested by the reduced production of effector cytokines including tumor necrosis factor alpha (TNF-α), interferon-γ, and IL-2 (Fig. 6*A*). In contrast, stable overexpression of *CWF19L1* augmented effector function of Jurkat cells (Fig. 6*B*). Consistent with this, knockdown of *CWF19L1* in Jurkat cells suppressed expression of the lytic molecule granzyme B (GZMB), whereas overexpression of *CWF19L1* promotes GZMB production in response to stimulation with phorbol 12-myristate 13-acetate and ionomycin (Fig. 6, *C* and *D*). These results support the notion that CWF19L1 plays a role in T-cell mediated cytotoxicity.

### CWF19L1 promotes T-cell mediated tumor killing

**Figure 7:**
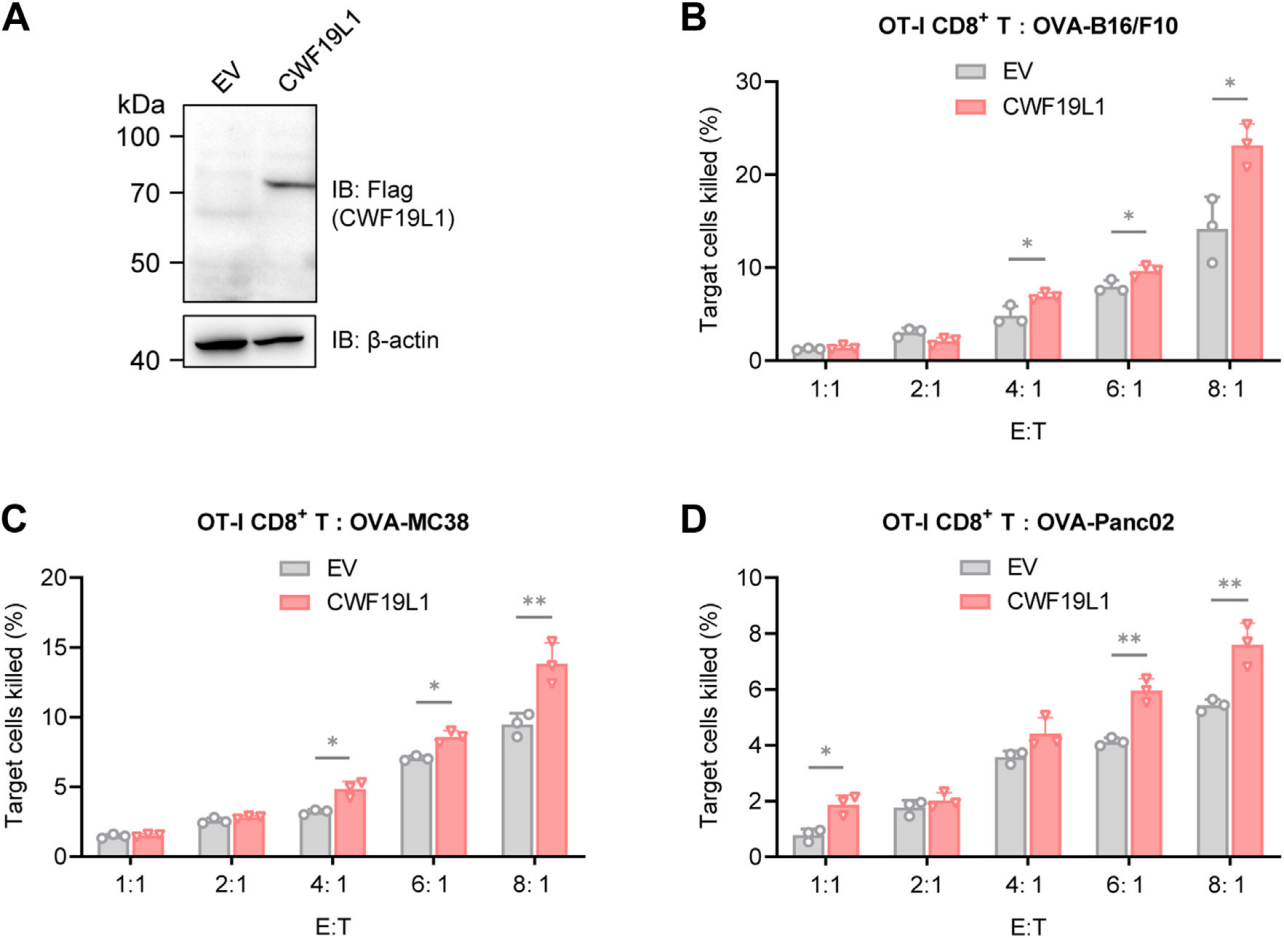
**CWF19L1 facilitates T-cell mediated tumor cell killing**. *A*, Western blot analysis of CWF19L1 expression in OT-I CD8^+^ T cells. Primary CD8^+^ T cells were isolated from the spleens of OT-I TCR transgenic mice using a negative selection protocol. The OT-I CD8^+^ T cells were stimulated with 2 μg/ml precoated anti-CD3 antibody, 1 μg/ml anti-CD28 antibody, and 10 ng/ml IL-2 for 24 h. Then, the OT-I CD8^+^ T cells were infected with retrovirus encoding CWF19L1 or nothing. Two days later, these T cells are harvested for cytotoxicity assay and Western blot analysis. *B*−*D*, percentage of target cells OVA-B16/F10 (*B*), OVA-MC38 (*C*), and OVA-Panc02 (*D*) killed by OT-I CD8^+^ T cells (described in A). Tumor cells were pulsed with OVA (257 − 264) peptides for 1 h and then plated into 96-well plates. The peptide-loaded B16/F10, MC38, and Panc02 cells were incubated with the OT-I CD8 T cells at different effector-to-target (E:T) ratios (1:1, 2:1, 4:1, 6:1, and 8:1). Apoptotic cells were detected by flow cytometry. *p* values were calculated using an unpaired Student′s *t* test. Data are presented as mean ± SD (∗*p* < 0.05, ∗∗*p* < 0.01). IL, interleukin; TCR, T cell receptor.

To further elucidate the role of CWF19L1 in the T cell response to antigen, we transduced CWF19L1 into OT-I T cell receptor (TCR) transgenic primary T cells. Subsequently, we evaluated the efficacy of CWF19L1-overexpressing CTLs in targeting murine B16/F10 melanoma cells, MC38 colon carcinoma cells, and Panc02 pancreatic tumor cells pulsed with ovalbumin peptides (OVA_257–264_). Remarkably, CWF19L1-overexpressing OT-I CTLs demonstrated significantly enhanced killing specificity against all three types of tumor cells compared to control OT-I CTLs (Fig. 7, *A*−*D*). These findings suggest that CWF19L1 enhances CTL cytotoxicity, thereby facilitating CD8^+^ T-cell mediated antitumor immunity.

## Discussion

The dynamic regulation of gene expression within cells requires highly precise and efficient splicing machinery. Central to this process is the spliceosome, a complex composed of five major snRNPs: U1, U2, U4, U5, and U6 snRNPs, along with numerous splicing-associated proteins (28, 29). In the current study, we first identified CWF19L1 as a potential splicing regulator. Mass spectrometry and Co-IP assays revealed that CWF19L1 interacts with key splicing factors and regulators, particularly components of the U5 snRNPs and the PRPF19 complex, indicating its integration into the splicing machinery. Deficiency of CWF19L1 resulted in significant abnormalities in alternative splicing, with SEs being the most frequent errors observed, underscoring its regulatory role in maintaining splicing fidelity. Consequently, these splicing defects led to alterations in global gene expression. Remarkably, CWF19L1 predominantly localizes to the nucleoplasm rather than specific nuclear substructures such as Cajal bodies or nuclear speckles. Cajal bodies are known sites for assembly of snRNPs, including the U4/U6.U5 tri-snRNP, which subsequently move to nuclear speckles where splicing factors are stored before being recruited for active transcription and pre-mRNA splicing (30). Our findings suggest that CWF19L1 may not directly participate in the assembly and storage of snRNPs within these specialized nuclear compartments. Notably, CWF19L1 exhibits the ability to shuttle between the nucleus and the cytoplasm, although its predominant steady-state localization is in the nucleoplasm (27). This dynamic behavior parallels that of U5 snRNPs, with which CWF19L1 interacts. U5 snRNPs also shuttle between the nucleus and cytoplasm, undergoing intricate biogenesis and ultimately exiting the spliceosome as a U5/PRPF19 post-splicing particle, ready for subsequent splicing cycles (31, 32, 33). Future research is needed to determine whether CWF19L1 shares a similar dynamic intracellular localization pattern with U5 snRNPs. Moreover, CWF19L1 was reported to interact with human Debranching enzyme 1 (hDbr1), which is involved in RNA lariat debranching (27). This interaction raises the possibility that CWF19L1 may also participate in post-splicing intron turnover. Further examination is required to explore this potential role. Overall, our study highlights CWF19L1 as a critical player in splicing regulation, influencing alternative splicing outcomes and thereby impacting gene expression patterns. Further investigations are also needed to elucidate the precise mechanisms by which CWF19L1 associates with splicing factors and modulates the splicing machinery.

CWF19L1 is highly conserved across different species and prominently expressed in immune organs. Additionally, CWF19L1 is ubiquitously expressed in T cells, including naïve CD8^+^T cells and effector CD8^+^T cells. Consistent with this expression pattern, the enrichment analyses of particular GO categories and KEGG pathways for CWF19L1-deficiency−induced differentially expressed genes indicated a potential role for CWF19L1 in regulating T cell cytotoxicity. CWF19L1 deficiency altered the splicing patterns of immune-related genes, resulting in reduced expression of genes involved in T cell cytotoxicity and cytokine production. Conversely, overexpression of CWF19L1 enhanced T cell effector functions and promoted secretion of effector cytokines upon activation. Moreover, CWF19L1 overexpression facilitated CTLs-mediated anti-tumor immunity, suggesting its potential as a target for immunotherapy to bolster immune responses against cancer. The mechanism behind the elevated levels of cytotoxic molecules following CWF19L1 modulation likely involves alternative splicing regulation, although detailed mechanistic insights remain to be fully elucidated. Of particular note, *CWF19L1* downregulation significantly altered the expression of several members of the TNF receptor superfamily (TNFRSF), including Fas cell surface death receptor (*FAS*), *TNFRSF10A*, *TNFRSF4*, and others. The TNF receptor superfamily is pivotal in the physiological regulation of programmed cell death (34, 35, 36). Given prior research suggesting that CWF19L1 is involved in regulating cell death and survival (25, 26), CWF19L1 may also influence cytotoxic effects mediated by apoptosis through its impact on the TNF signaling pathways. Furthermore, CWF19L1 deficiency altered the expression of genes involved in NK cell-mediated killing, T cell differentiation, and inflammatory responses, indicating broader implications for antitumor immune responses beyond T cell cytotoxicity.

In conclusion, our study elucidates CWF19L1 as a crucial regulator within the splicing machinery, influencing alternative splicing fidelity and gene expression dynamics which are critical for immune functions such as T cell cytotoxicity. These findings lay the groundwork for future research aimed at unraveling the mechanistic underpinnings of CWF19L1-mediated immune modulation and exploring its therapeutic potential in immune-related disorders and cancer immunotherapies.

## Experimental procedures

### Antibodies

Primary antibodies used for immunoblotting and immunofluorescence were: anti-CWF19L1 (Cat.#D124694) from Sangon Biotech; anti-CDC5L (Cat.# sc-398280), anti-BCAS2 (Cat.# sc-376554), anti-PUF60 (Cat.# sc-398799), anti-RNPC3 (Cat.# sc-514951), anti-PPIH (Cat.# sc-377217), anti-Coilin (clone F-7, Cat.# sc-55594), anti-p54/nrb (clone G-1, Cat.# sc-376865), anti-Fibrillarin (clone G-8, Cat.# sc-374022), anti-promyelocytic leukemia (clone PG-M3, Cat.# sc-996), and anti-p-SC35/SRSF2 (clone SC-35, Cat.# sc-53518) from Santa Cruz Biotechnology; anti-SNRNP40 (Cat.# HPA026527) from Atlas Antibodies; anti-EFTUD2 (Cat.# A7040), anti-AAR2 (Cat.# A18443), anti-PRPF4 (Cat.# A21132), anti-PRPF31 (Cat.# A20979), anti-PRPF19 (Cat.# A12590), anti-SART1 (Cat.# A8569), anti-SNRPA1 (Cat.# A6410), anti-PQBP1 (Cat.# A4369), anti-PRPF6 (Cat.# A6053), anti-SNRPB (Cat.# A2009), anti-U2AF2 (Cat.# A1936), anti-SF3B1 (Cat.# A9737), and anti-U2AF1 (Cat.# A13166) from ABclonal; anti-β-actin (Cat.# 60008-1-Ig) from Proteintech; anti-FLAG (clone M2, Cat.#F1804) from Sigma−Aldrich. Secondary antibodies used for immunoblotting were Peroxidase-conjugated AffiniPure Goat Anti-Rabbit immunoglobulin G (IgG) (H+L) (Cat.# 111–035–003) and Peroxidase-conjugated AffiniPure Goat Anti-Mouse IgG (H+L) (Cat.# 115–035–003) from The Jackson Laboratory. Secondary antibody used for immunofluorescence was DyLight 594 anti-Mouse IgG (Cat.# 35510) from Invitrogen. Antibody used for Flow cytometry was PE anti-granzyme B (clone QA16A02, Cat.# 372207) from BioLegend. All antibodies used in the study were validated for species and application by the vendors. Validation data are available on the manufacturer's websites (checkable *via* the category information above). We further validated the primary antibodies used in immunoblotting by their molecular weight or using negative samples.

### Cell lines and culture

HeLa, A549, Panc02, Jurkat, MC38, B16/F10, and HEK293T cells were purchased from the American Type Culture Collection. HeLa, A549, Panc02, MC38, and HEK293T cells were maintained in Dulbecco′s modified Eagle′s medium. B16/F10 and Jurkat cells were cultured in RPMI-1640 medium. All media were supplemented with 10% fetal bovine serum, 100 U/ml of penicillin, and 100 μg/ml of streptomycin. Cells were cultured in a humidified incubator at 37 °C with 5% CO2. There were no signs of *mycoplasma* contamination in any of these cell lines. Cell line authentication was performed *via* short tandem repeat profiling.

### Animals

OT-l TCR transgenic mice were generously provided by Dr Bing Du from East China Normal University. The mice were housed under specific-pathogen-free conditions in the animal facility at Fudan University. Environmental controls in the animal room included a temperature range of 18 − 23 °C, humidity levels of 40 − 60%, and a 12-h light/12-h dark cycle. The study was approved and monitored by the Institutional Committee for Animal Welfare of Fudan University, and all animal procedures were conducted in accordance with the institution′s guidelines.

### Plasmids and shRNA constructs

The plasmid expressing Flag-tagged CWF19L1 was generated by inserting the human *CWF19L1* coding sequence into the pcDNA6-N-Flag vector. The pCMV-E1A construct was generated by inserting a fragment of E1A complementary DNA (cDNA) (nucleotides 533–1341) from the Adenovirus genome into the EcoRI/NotI sites of pCMV vector. The retroviral plasmid encoding Flag-tagged CWF19L1 was generated by inserting the mouse *Cwf19l1* coding sequence into the pMSGV-N-Flag vector. Plasmids for knockdown were constructed by inserting annealed shRNAs into the pLV-H1-EF1α-puro lentiviral vector. The shRNA sequences specifically targeting *CWF19L1* were 5′-GGAACTTGCCTACAGCAAGAG-3′ (sh*CWF19L1*#1) and 5′-GGATGCTGATGGATGTGAATT-3′ (sh*CWF19L1*#2). The nontargeting control shRNA (shCtrl) sequence was 5′-CAACAAGATGAAGAGCACC-3′. All constructs were veriﬁed by DNA sequencing. Primers are listed in Table S1.

### Lentiviral and retrovirus production and infection

Lentivirus was produced in HEK293T cells by cotransfecting the lentiviral vector with the packaging plasmids (pMDLg/pRRE, VSV-G, and pRSV-REV) using calcium phosphate-mediated transfection. Retrovirus was produced similarly by transfecting HEK293T cells with the retroviral vector and the packaging plasmid (pCL-Eco). Stable cell lines were established through lentivirus or retrovirus infection, followed by puromycin selection.

### Coimmunoprecipitation and mass spectrometry analysis

HEK293T cells transfected with Flag-tagged CWF19L1 or empty vector were harvested in NP-40 lysis buffer (25 mM Tris–HCl, pH 7.4; 150 mM NaCl; 1% NP-40; 1 mM EDTA; 1 mM EGTA; 1 × protease, and phosphatase inhibitor cocktail), and incubated on ice for 30 min followed by sonication for 15 s. Cell lysates were centrifuged at 12,000*g* for 10 min, and 10% of the supernatants were saved for preparing the input samples. The other supernatants were immunoprecipitated with anti-Flag M2 magnetic beads (Sigma−Aldrich) for 3 h at 4 °C on a rotor. After immunoprecipitation, beads were washed extensively, and agarose-bound proteins were eluted using 3 × FLAG peptide (Sigma−Aldrich) and analyzed by immunoblotting. To identify CWF19L1-interacting partners, the eluted proteins were subjected to silver staining and semiquantitative mass spectrometry (liquid chromatography−tandem mass spectrometry) analysis by the Proteomics Core Facility at the Institutes of Biomedical Sciences, Fudan University. Total and top CWF19L1-interacting candidates are listed in Dataset S1.

### Endogenous immunoprecipitation

A549 cells grown in three 10-cm dishes were harvested for protein extraction using NP-40 lysis buffer. The lysates were clarified by centrifugation, and the supernatants were precleared with Protein A/G agarose beads for 2 h at 4 °C. The precleared lysates were split into two equal aliquots and incubated overnight at 4 °C with 3 μg of either anti-CWF19L1 antibody or control IgG (Santa Cruz Biotechnology) under gentle agitation. The following day, Sera-Mag SpeedBeads Protein A/G Magnetic beads (Cytiva) were washed and equilibrated in wash buffer (25 mM Tris–HCl, pH 7.5, 0.65 M NaCl, and 0.05% Tween-20) before being added to the antibody-bound lysates. The mixtures were incubated for 1 h at room temperature (RT) with continuous mixing. Immunocomplexes were eluted using a low-pH elution buffer (0.1 M glycine, pH 2.5), and the eluates were immediately neutralized by adding 1/10 volume of 1 M Tris–HCl, pH 9.0. The samples were then subjected to Western blot analysis.

### Western blot

Proteins in the cell lysates were separated by SDS-PAGE and transferred onto nitrocellulose membranes. The membranes were blocked with 5% nonfat milk in Tris-buffered saline to minimize nonspecific binding. Following blocking, the membranes were incubated with the appropriate primary antibodies and subsequently with horseradish peroxidase-conjugated secondary antibodies for 1 h at RT. Protein detection was performed using an electrochemiluminescence system according to the manufacturer′s protocol.

### RNA-seq and data analysis

Total RNA was isolated from *CWF19L1* knockdown and control cells using TRIzol reagent (Invitrogen) according to the manufacturer′s protocol. Subsequently, 1 μg total RNA was used for the following library preparation. Poly(A) mRNA isolation was performed using Oligo(dT) beads. The mRNA fragmentation was performed using divalent cations and high temperature. Priming was performed using random primers. First-strand cDNA and the second-strand cDNA were synthesized. The purified double-stranded cDNA was then treated to repair both ends and add a dA tailing in one reaction, followed by T-A ligation to add adaptors to both ends. Size selection of adaptor-ligated DNA was then performed using DNA Clean Beads. Each sample was then amplified by PCR using P5 and P7 primers, and the PCR products were validated. Then libraries with different indices were multiplexed and loaded on an Illumina HiSeq, Illumina Novaseq, or MGI2000 instrument for sequencing using a 2 × 150 paired-end configuration according to the manufacturer′s instructions.

Pass filter data in fastq format were processed by Cutadapt (v1.9.1, phred cutoff: 20, error rate: 0.1, adapter overlap: 1 bp, min. length: 75, proportion of N: 0.1) (https://cutadapt.readthedocs.io/en/stable/) to obtain high-quality clean data. Clean data were aligned to reference genome *via* software Hisat2 (v2.2.1) (https://ccb.jhu.edu/software/hisat2/). Expression analysis used the HTSeq (v0.6.1) (https://htseq.readthedocs.io/en/release_0.6.1/). Differential expression analysis used the DESeq2 Bioconductor (https://bioconductor.org/packages/DESeq2/) package, a model based on the negative binomial distribution. The estimates of dispersion and logarithmic fold changes incorporated data-driven prior distributions, Padj values of genes were set to ≤0.05 to detect differentially expressed ones. GOSeq (v1.34.1) (https://bioconductor.org/packages/release/bioc/html/goseq.html) was used to identify GO terms that annotate a list of enriched genes with a significant Padj value less than or equal to 0.05. KEGG is a collection of databases dealing with genomes, biological pathways, diseases, drugs, and chemical substances (http://en.wikipedia.org/wiki/KEGG). Novel transcripts can be predicted using the results of Cufflinks v2.2.1. Alternative splicing analysis was performed using rMATs v4.1.0. Samtools v0.1.19 (https://sourceforge.net/projects/samtools/files/samtools/0.1.19/) with command mpileup and Bcftools v0.1.19 were used to do SNV calling. To analyze DEU, we used the Bioconductor package DEXSeq (https://bioconductor.org/packages/release/bioc/html/DEXSeq.html). The alternative splicing events and differentially expressed genes are listed in Dataset S2 and S3, respectively.

### Reverse transcription polymerase chain reaction

Total RNA was isolated using TRIzol reagent (Invitrogen). Additionally, 1 μg of total RNA was reverse transcribed to cDNA. RT−PCR was conducted on 1 μl of cDNA using 2 × Taq Plus Master Mix (Vazyme) to confirm splicing and splicing modulation. Forty-eight hours after transfection with pCMV-E1A, total RNA from independent duplicate transfections was extracted, treated with DNase I, and analyzed for E1A splicing by RT−PCR. PCR conditions were as described in the manufacturer′s protocol. The list of primers used for RT−PCR in this study is provided in Table S1.

### Quantitative real-time PCR (RT‒qPCR)

Total RNA was extracted using TRIzol reagent (Invitrogen). Subsequently, 1 μg of total RNA was reverse transcribed into cDNA. RT−qPCR was performed on the cDNA using 2 × SYBR Green PCR Master Mix (Vazyme) and a QuantStudio 1 (Applied Biosystems). Fold changes were calculated by relative quantification. Primers used for RT−qPCR are listed in Table S1.

### Immunofluorescence

Cells grown on coverslips were fixed with 4% paraformaldehyde for 10 min at RT. The coverslips were permeabilized in 0.2% Triton X-100/PBS for 10 min on ice, followed by blocking in 1% bovine serum albumin/PBS for 15 min. Primary antibodies were applied at a 1:100 dilution in 1% bovine serum albumin/PBS and incubated overnight at 4 °C. After washing with PBS, the coverslips were stained with secondary antibodies at a 1:200 dilution for 1 h at RT in the dark. Nuclei were visualized with the DNA-specific dye 4′,6-diamidino-2-phenylindole. Specimens were analyzed using an LSM900 Zeiss confocal microscope system.

### Flow cytometry

For cytokine production analysis, CWF19L1-depleted or overexpressing Jurkat T cells were seeded in a round-bottom 96-well plate and stimulated with 50 ng/ml phorbol 12-myristate 13-acetate and 1 μg/ml ionomycin for various time periods. Then, the cells were fixed and permeabilized, followed by staining with paired-end anti-granzyme B (Invitrogen, MHGB04) for 40 min at 4 °C in the dark. After washing, cells samples were analyzed using a FACSCelesta Flow Cytometer (BD Biosciences) and FlowJo software (version 10.8.1) (https://www.flowjo.com/).

### OT-I T cell killing assay *in vitro*

OT-I CD8^+^ T cells were isolated from the spleen of OT-I TCR transgenic mice using a negative selection kit (BioLegend, 480008) according to the manufacturer′s instructions. A total of 1 × 10∧6 CD8^+^ T cells per well were seeded in a 24-well F-bottom plate, precoated with anti-CD3 antibody (2 μg/ml; BioLegend, 100340). The cells were cultured in RPMI-1640 complete medium supplemented with 1 μg/ml anti-CD28 antibody (BioLegend, 102116) and 10 ng/ml interleukin 2 (Novoprotein, C013). After 24 h of stimulation, the OT-I CD8^+^ T cells were infected with a retrovirus encoding CWF19L1 expression. Tumor cells were pulsed with OVA_257–264_ peptides (Sangon Biotech, T510212) for 1 h and then plated into 96-well plates at a density of 2 × 10∧4 cells per well. OT-I CD8^+^ T cells were collected and incubated with the targets (OVA-B16/F10 cells, OVA-MC38, and OVA-Panc02) at different E:T ratios for 2 h. Cells were stained with FITC anti-mouse CD8a (BioLegend, 100706) for T cells and propidium iodide (BD Biosciences, 51–66211E) for apoptotic tumor cells, followed by flow cytometric analysis.

## Data availability

All data are contained within the article and the Supporting Information. The RNA-seq data presented in this study were deposited into the GEO repository (accession number GSE273485).

## Supporting information

This article contains supporting information.

## Conflict of interest

The authors declare that they have no conflicts of interest with the contents of this article.
