# Supplementary material for: CWF19L1 promotes T-cell cytotoxicity through the regulation of alternative splicing
Source: J Biol Chem. 2024 Nov 13;300(12):107982. doi: 10.1016/j.jbc.2024.107982 (PMC11665689; doi:10.1016/j.jbc.2024.107982)
Supplement: Supplementary Fig. S1-S6 [file mmc4.pdf]

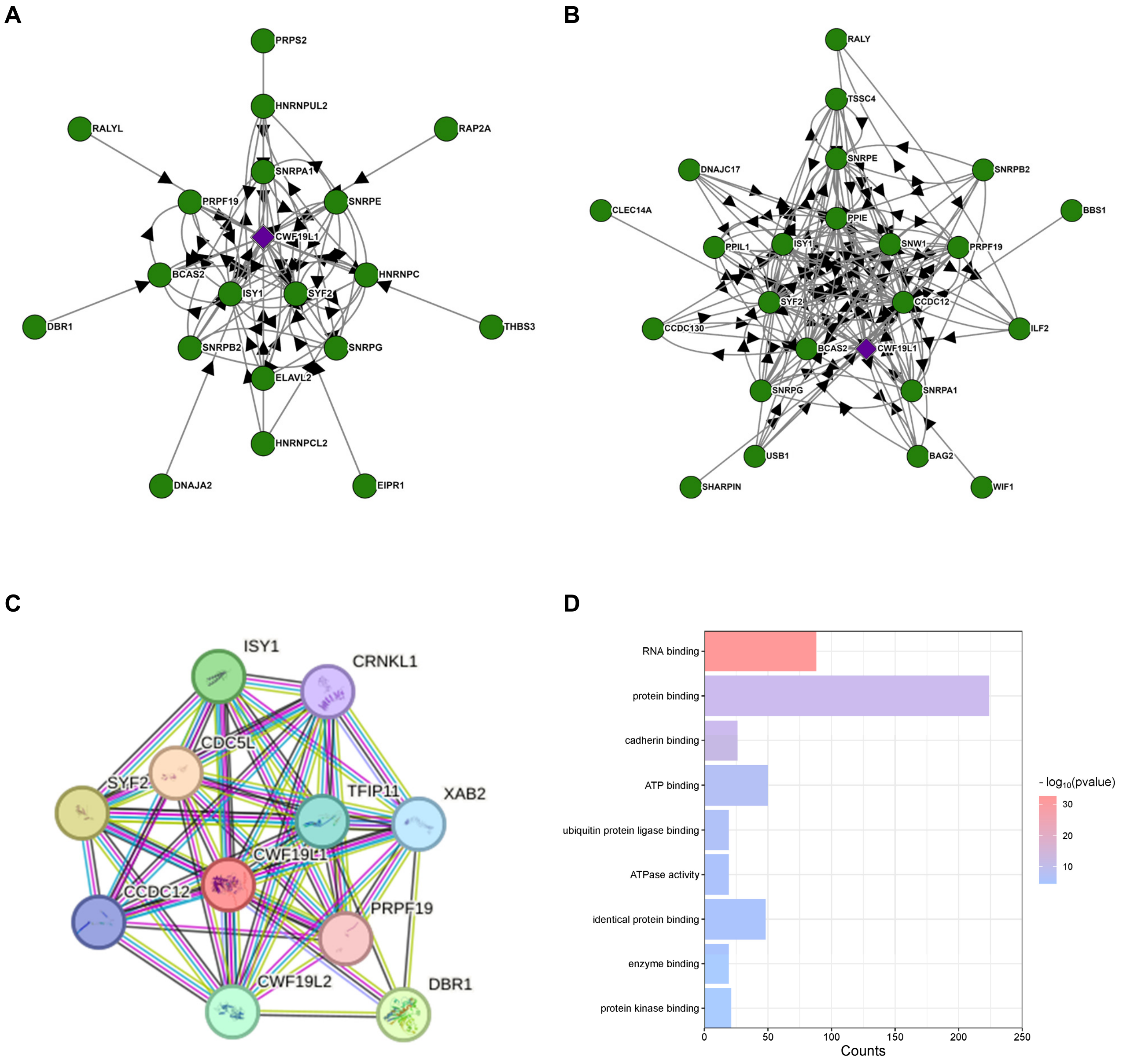

Figure S1. CWF19L1-interacting protein networks.

(**A** and **B**) CWF19L1-interacting candidates identified in HEK293T cells (**A**) and HCT116 cells (**B**) according to the BioPlex dataset. The majority of the candidates are splicing-related proteins.

(**C**) CWF19L1-associated proteins as revealed by STRING protein-protein interaction networks (version 12.0).

(**D**) GO molecular function analysis of CWF19L1-interacting proteins identified in the Co-IP/MS experiment.

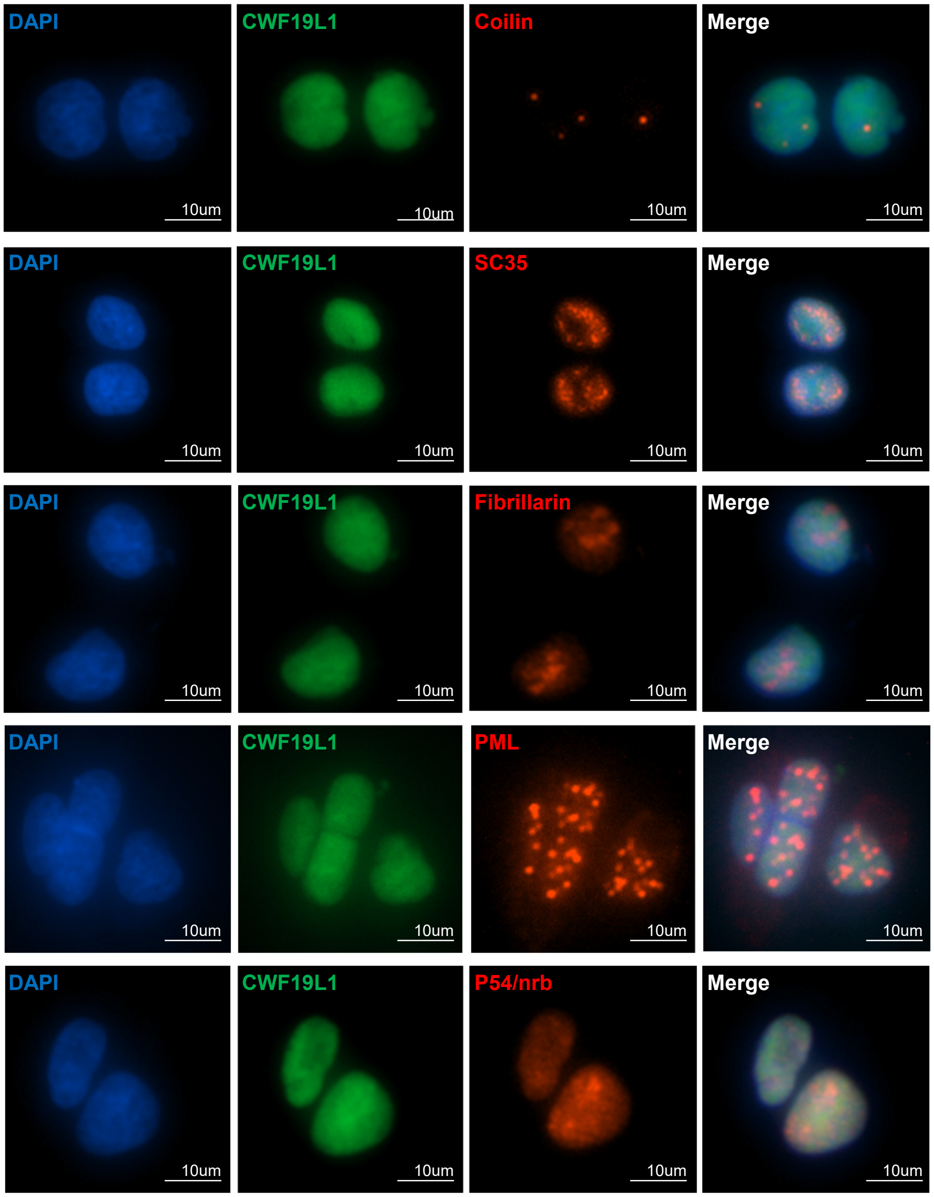

Figure S2. Subcellular localization of CWF19L1.

HeLa cells transfected with a plasmid encoding the EGFP-CWF19L1 fusion protein (green) were immunostained with antibodies against various subnuclear markers: anti-Coilin (Cajal body), anti-SC35 (nuclear speckle), anti-PML (PML-nuclear body), anti-Fibrillarin (nucleoli), and anti-p54/nrb (NONO, paraspeckle). Immunofluorescence signals were detected using a DyLight 594 anti-Mouse IgG secondary antibody (red). Nuclei were counterstained with DAPI (blue).

A

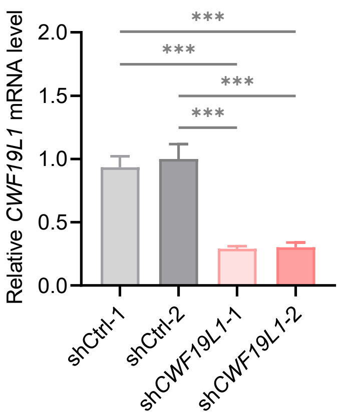

B

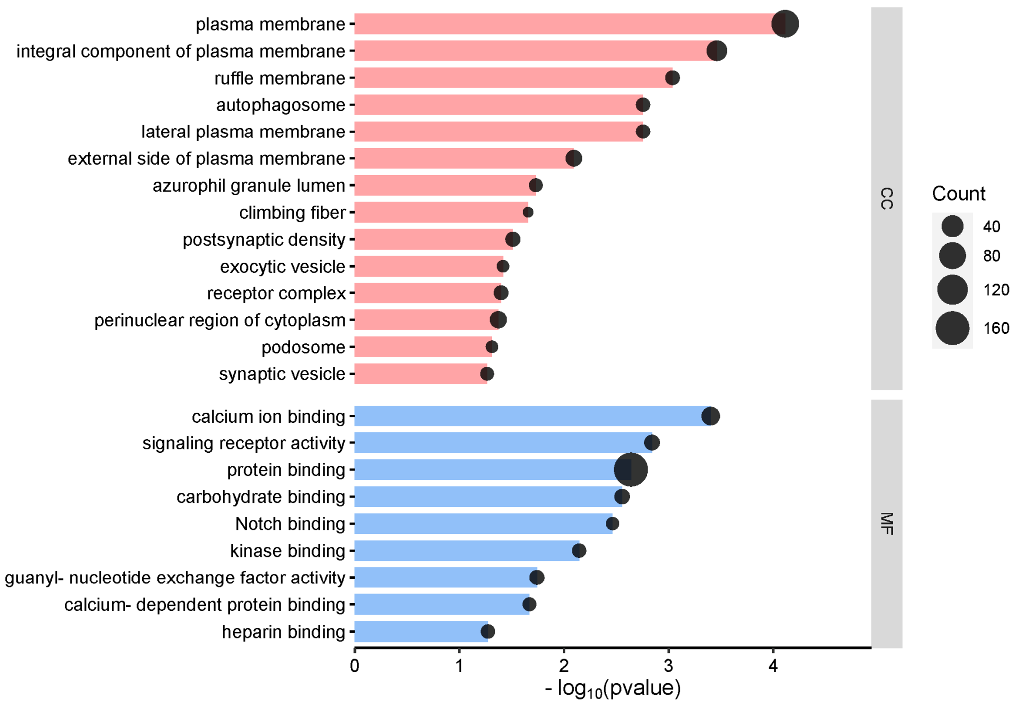

Figure S3. Enrichment analysis of differentially expressed genes in CWF19L1-deficent and control cells revealed by RNA-seq.

(**A**) RT-qPCR analysis of expression levels of CWF19L1 in HEK293T cells infected with lentivirus encoding shRNA against CWF19L1 (sh*CWF19L1*) or nontargeting control shRNA (shCtrl). These cells were then transfected with E1A reporter minigene to assess alternative splicing. P values were calculated using an unpaired Student's *t*-test between two groups. Data are presented as mean ± SD. ****P* < 0.001.

(**B**) GO cellular components (top) and molecular function (bottom) analysis of differentially expressed genes in CWF19L1-depleted versus control Jurkat cells revealed by RNA-seq.

Percent Identity Matrix

sp|Q8CI33|C19L1_MOUSE	100.00%	95.90%	89.31%	89.39%	89.31%	90.13%	90.88%	91.43%
tr|A6JHE9|A6JHE9_RAT	95.90%	100.00%	89.87%	89.94%	90.06%	90.50%	91.62%	91.99%
tr|A0A2K6L656|A0A2K6L656_RHIBE	89.31%	89.87%	100.00%	95.88%	96.82%	93.26%	94.19%	95.32%
sp|Q69YN2|C19L1_HUMAN	89.39%	89.94%	95.88%	100.00%	98.31%	93.12%	94.24%	95.54%
tr|A0A2I3SDJ1|A0A2I3SDJ1_PANTR	89.31%	90.06%	96.82%	98.31%	100.00%	92.70%	94.01%	95.13%
tr|A0A2Y9N695|A0A2Y9N695_DELLE	90.13%	90.50%	93.26%	93.12%	92.70%	100.00%	94.80%	95.35%
tr|E1BG07|E1BG07_BOVIN	90.88%	91.62%	94.19%	94.24%	94.01%	94.80%	100.00%	97.40%
tr|A0A4X1U4C8|A0A4X1U4C8_PIG	91.43%	91.99%	95.32%	95.54%	95.13%	95.35%	97.40%	100.00%

Figure S4. CWF19L1 protein is conversed in multiple mammalian species.

Homology alignment of the CWF19L1 amino acid sequence across multiple species. The alignment was performed using UniProt.

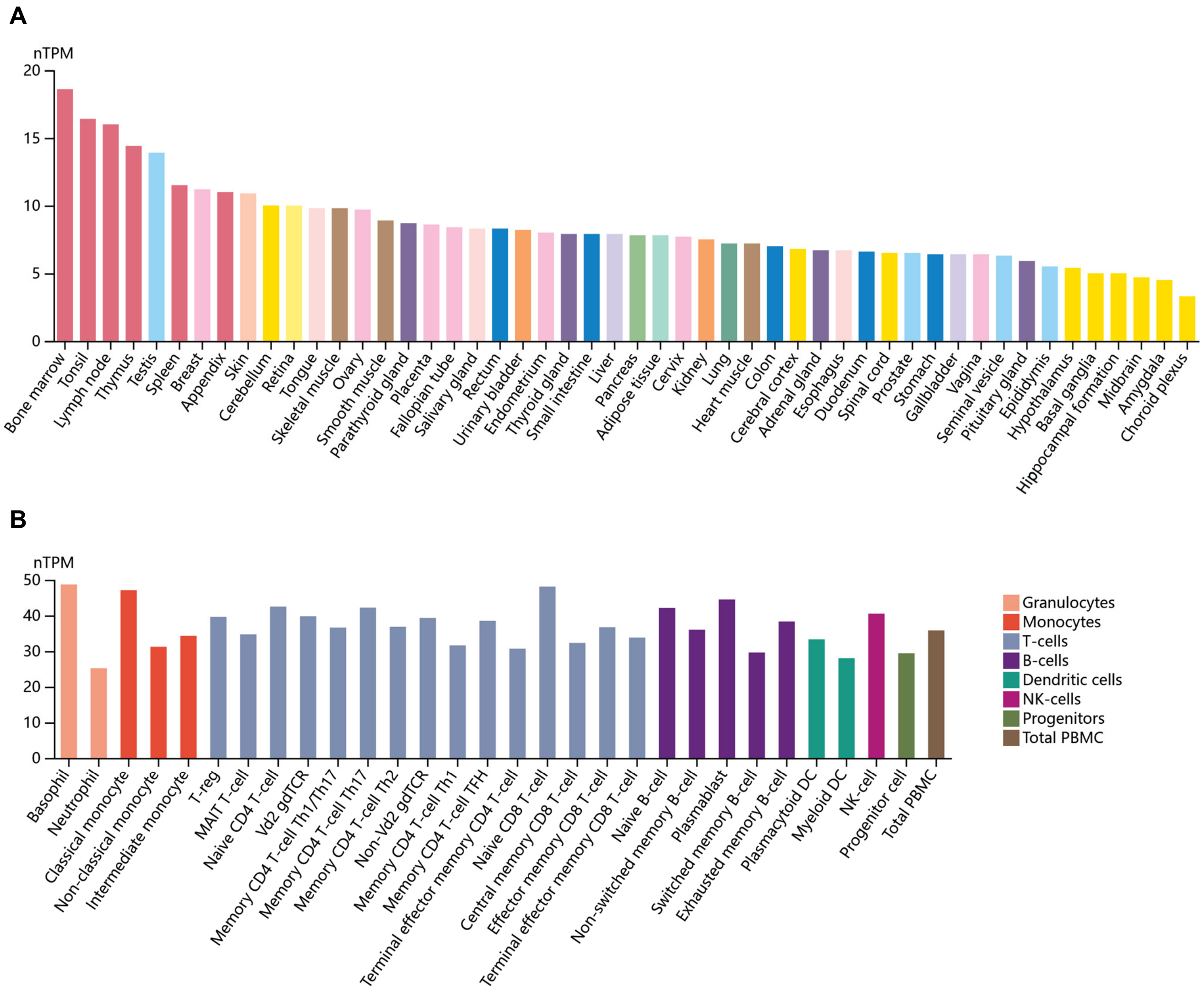

Figure S5. CWF19L1 expression profiles in various tissues and immune cell types.

(**A**) Consensus normalized expression levels of CWF19L1 in 55 tissue types, created by combining the Human Protein Atlas (HPA) and GTEx transcriptomics datasets using the internal normalization pipeline by HPA. The data are reported as nTPM (normalized protein-coding transcripts per million). Color-coding is based on tissue groups, each consisting of tissues with functional features in common.

(**B**) The transcript expression levels of CWF19L1 in 29 blood cell types and total peripheral blood mononuclear cells (PBMC) from the Monaco dataset.

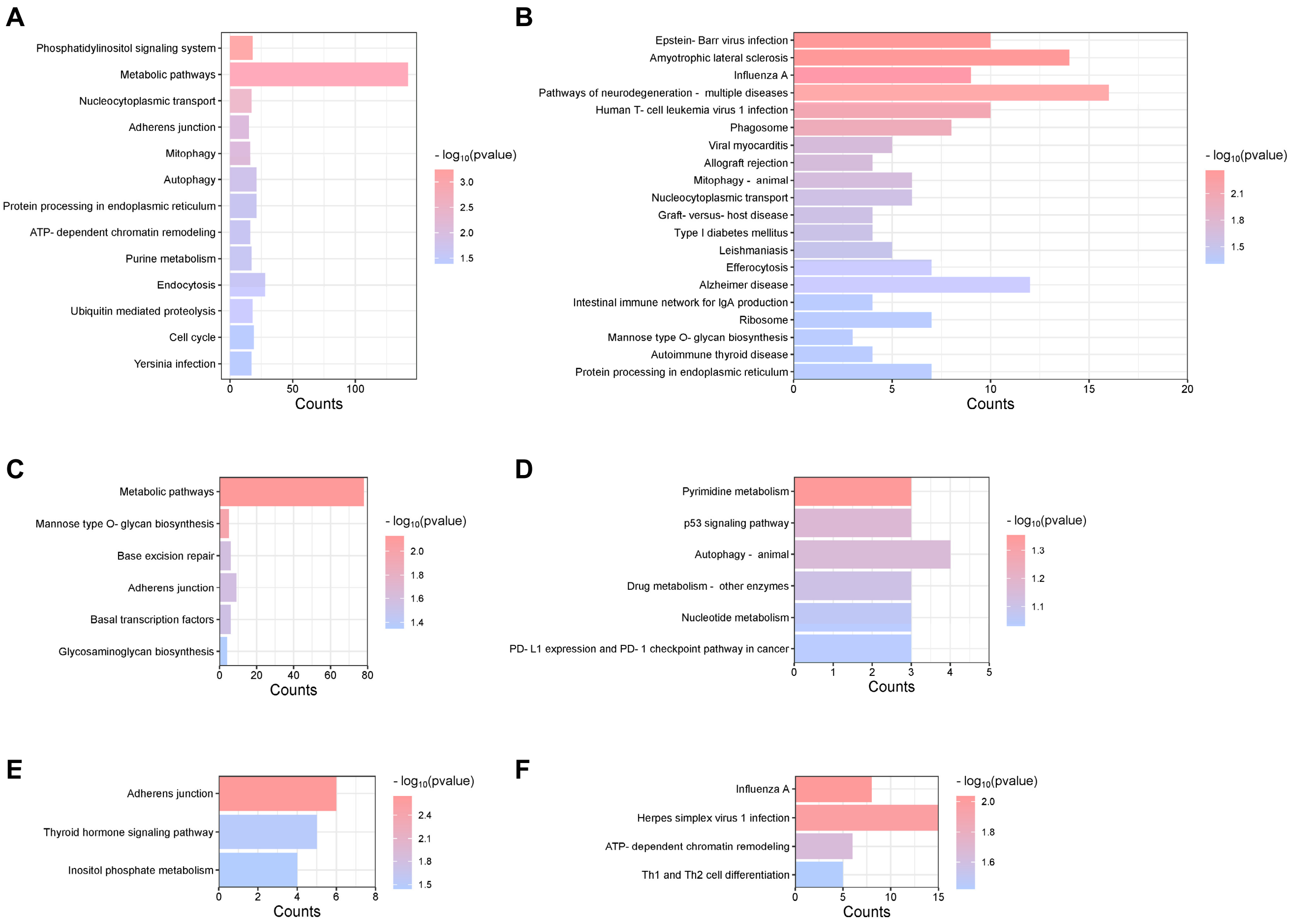

Figure S6. Enrichment analyses of alternative splicing abnormalities and differentially expressed immune-related genes.

(**A**) KEGG pathway analysis of alternative splicing abnormalities induced by CWF19L1 deficiency. The ordinate represents the pathway name, the abscissa represents the number of genes enriched in each pathway, and the color of the column indicates the p-value.

(**B**−**F**) KEGG pathway analyses of different types of alternative splicing abnormalities (**B**, RI; **C**, SE; **D**, MXE; **E**, A5SS; **F**, A3SS).
