# Supplementary material for: CWF19L1 promotes T-cell cytotoxicity through the regulation of alternative splicing
Source: J Biol Chem. 2024 Nov 13;300(12):107982. doi: 10.1016/j.jbc.2024.107982 (PMC11665689; doi:10.1016/j.jbc.2024.107982)
Supplement: Table S1 [file mmc5.pdf]

Table S1. Oligonucleotides used in the study, not including subcloning primers.

RT-qPCR primers	Sequence (5′ − 3′)	RT-PCR primers	Sequence (5′ − 3′)
ACTB-F	ACCTTCTACAATGAGCTGCG	*E1A-F*	ATTATCTGCCACGGAGGTGT
ACTB-R	CCTGGATAGCAACGTACATGG	*E1A-R*	GGATAGCAGGCGCCATTTTA
IL-2-F	AAAGAAAACACAGCTACAACTGG	*ACTB-RT-F*	ACCTTCTACAATGAGCTGCG
IL-2-R	GAAGATGTTTCAGTTCTGTGGC	*ACTB-RT-F*	CCTGGATAGCAACGTACATGG
IFNG-F	GCATCGTTTTGGGTTCTCTTG	*TNFRSF4-RI-Intron2-F*	CAGAACACGGTGTGCCGTC
IFNG-R	AGTTCCATTATCCGCTACATCTG	*TNFRSF4-RI-Intron2-R*	CTCCAGGCTTGTAGCTGTCCAG
GZMB-F	GTACCATTGAGTTGTGCGTG	*TNFRSF4-RI-Intron5-F*	CATCACTGTCCAGCCCACTGAAG
GZMB-R	CATGCCATTGTTTCGTCCATAG	*TNFRSF4-RI-Intron5-R*	GGTACAGGGCCAGCAGGATG
TNFA-F	ACTTTGGAGTGATCGGCC	*IL4R-SE-Exon10-F*	GAATGGTGGGATCAGATTCCCAACC
TNFA-R	GCTTGAGGGTTTGCTACAAC	*IL4R-SE-Exon10-R*	CTCTGGCCAGAGGACTGTCTTG
TNFRSF4-F	AAGCCTGGAGTTGACTGTG	*SHMT1-SE-Exon8-F*	TCTCATGGCGGACATGGCTC
TNFRSF4-R	GTCCTCACAGATTGCGTCC	*SHMT1-SE-Exon8-R*	CTGTGACTATTTTGTAGCCCAGCTCC
RORC-F	TGGTGCTGGTTAGGATGTG		
RORC-R	GGAGTGGGAGAAGTCAAAGATG		
MAML3-F	AGCAGAGGGCCAAGAAATC		
MAML3-R	CTCCTTCCAACTTCCTTTTCAC		
HEG1-F	AACATCTCCTTCTCCCCAAAC		
HEG1-R	AGACAAGGGTTCACAGCAC		
IL1R2-F	AGTTTTCAGACACTACGCACC		
IL1R2-R	CCATCTGCTTTTCCAGTTCTG		
TNFRSF10A-F	CAGTGCAAACCAGGAACTTTC		
TNFRSF10A-R	GTCCATTGCCTGATTCTTTGTG		
IL2RB-F	ACCCCTCGAAGTTCTTTTCC		
IL2RB-R	TCACCTTGTCCCTCTCCAG		
CD244-F	TCTAAGCGCACTGTTCCTTG		
CD244-R	CCTGCTCGTGATTTCTCCTG		
IL17RE-F	AATTCCTTCTGCCCTGTCTG		
IL17RE-R	TCCAGAAGTCCGAGCCATAG		
MAML2-F	GTGGGATAAACGGAGAGCAG		
MAML2-R	TGTTGGCAGGAGATAGGTTAAC		
MMRN2-F	ATTCCATGGCAATCCCTGAG		
MMRN2-R	AGATGTTCCTGCTGTTCCTG		
ITGAL-F	GACAACTCAGCCACTACCATC		
ITGAL-R	ACCTGGTACATGTGCTTGAC		
